# Lower pass threshold (≥93%) for critical congenital heart disease screening at high altitude prevents repeat screening and reduces false positives

**DOI:** 10.1038/s41372-022-01491-6

**Published:** 2022-08-17

**Authors:** M. Rhonda Sneeringer, Pranjali Vadlaputi, Satyan Lakshminrusimha, Heather Siefkes

**Affiliations:** 1 Barton Memorial Hospital, South Lake Tahoe, CA USA; 2 Department of Pediatrics, University of California, Davis, Sacramento, CA USA

**Keywords:** Outcomes research, Scientific community

## Abstract

**Objective:**

We evaluated first screen pass rate for two pass thresholds for critical congenital heart disease (CCHD) oxygen saturation (SpO_2_) screening at higher altitude.

**Study design:**

A retrospective cohort of 948 newborns underwent CCHD screening near sea-level (*n* = 463) vs 6250 ft altitude (*n* = 485) over 3 years. Standard SpO_2_ pass threshold ≥95% and lower SpO_2_ pass threshold ≥93% (high-altitude screen) were applied to first measurements to compare pass frequencies.

**Results:**

The median SpO_2_ was lower in high-altitude newborns (96% vs 99%—*p* < 0.001). The high-altitude newborns passed the AAP algorithm first screen less often (89.3% vs 99.6%—*p* < 0.001). With the high-altitude algorithm, 98% of high-altitude newborns passed the first screen.

**Conclusion:**

Lowering the SpO_2_ pass threshold by 2% at >6000 ft, significantly increased first screen pass rate. Adjustments for altitude may reduce nursing time to conduct repeat measurements and prevent transfers for echocardiograms. Larger studies are necessary to assess impact on false negatives.

## Introduction

Critical congenital heart disease (CCHD) screening using pulse oximetry was recommended by the US Health and Human Services Secretary’s Advisory Committee on Heritable Disorders in Newborns and Children and was added to the recommended uniform screening panel [1]. The American Academy of Pediatrics (AAP) workgroup provided an algorithm for universal screening of newborns [2]. A Cochrane review of CCHD screening using similar oxygen saturation (SpO_2_) thresholds as the recommended AAP algorithm showed a low false positive rate of 0.14%. The false positive rate was lower (0.06%) when the screen was completed after 24 h after birth [3]. However, few studies have evaluated the performance of the screening at high altitude (>6000 ft) [4, 5]. The American Academy of Pediatrics (AAP) recommends that “algorithm cutoffs may need to be adjusted in high-altitude nurseries.” [2] However, no specific approach is specified for high-altitude CCHD screening.

More recently, studies have recognized that modifications to this algorithm are needed at high altitude to reduce the frequency of screening failures [6, 7]. Barton Memorial Hospital in South Lake Tahoe, California (elevation > 6000 ft) implemented a modified algorithm using a threshold of ≥93% (instead of ≥95% in the AAP algorithm) as their criterion to pass CCHD screening without prompting repeat measurements due to their initial experience with false positives with a higher threshold. We hypothesized, based on physiologic data (Table 1), this threshold at ≥93% for a passing screen at higher altitude would result in higher first pass screen compared to the standard threshold of ≥95% without need to prompt repeat measurement.

**Table 1:** Calculation of change in oxygen pressure and saturation at 6000 ft altitude.

Altitude	Sea level (30 ft)	6000 ft
Atmospheric pressure	760 mmHg	609 mmHg
Partial pressure of water (*P*_H2O_)	47 mmHg	47 mmHg
Dry air atmospheric pressure	713 mmHg	562 mmHg
Partial pressure of inspired oxygen (PIO_2_)	150 mmHg	118 mmHg
Alveolar oxygen (PAO_2_)	107 ± 6.2 mmHg [15]	75 mmHg
Arterial oxygen (PaO_2_)	77 ± 4.5 mmHg [23] to 78.7 ± 10.4 mmHg [15]	45–56 mmHg^a^
Pulse oximetry (SpO_2_)	99% (IQR 98–100) [24]	95 (IQR 94–96)% [25]

*IQR* interquartile range.

^a^Alveolar to arterial gradient (A-a gradient) decreases with altitude [16].

## Methods

This was a retrospective cohort review of newborns undergoing routine SpO_2_-based CCHD screening at two altitudes in Northern California. University of California, Davis in Sacramento was the near-sea level site (30 ft elevation from sea level) and Barton Memorial Hospital in South Lake Tahoe, California was the high-altitude site (6250 ft elevation). University of California, Davis Institutional Review Board approved this study for both sites.

We estimated that 3 years of data would be necessary for adequate sample size from the high-altitude site (see analysis for sample size). Thus, we included patients of the same time period from the lower altitude site, January 2016 to December 2018. Due to higher birth rate at the lower altitude site, we included select patients to result in similar number of patients at each site. To select patients from the lower altitude site, we sorted by alphabetical order and then selected every 7th patient to ensure there was not a chronological pattern to the selection.

Protocols for CCHD screening were standardized at each site during these time periods. Patients that underwent routine SpO_2_ CCHD screening were included. Both sites performed SpO_2_ CCHD screening after 24 h of age or just before discharge if a newborn was discharged before 24 h. Subjects were excluded if they were admitted to the Neonatal Intensive Care Unit (NICU), transferred, or had an echocardiogram completed before completion of the routine SpO_2_ CCHD screening. Patients that were admitted to the NICU, transferred or had echocardiogram completed following the routine SpO_2_ CCHD screen were included since the results of the screen could lead to these interventions. They were also excluded if SpO_2_ screening results with numeric values were not available. Electronic medical records were reviewed for SpO_2_ screening results (interpretation and SpO_2_ values), medical conditions, echocardiograms and procedures. To identify potentially false negative screens, follow up encounters within the medical system, at least 6 weeks after birth, were reviewed for evidence of cardiac disease.

**Fig. 1:**
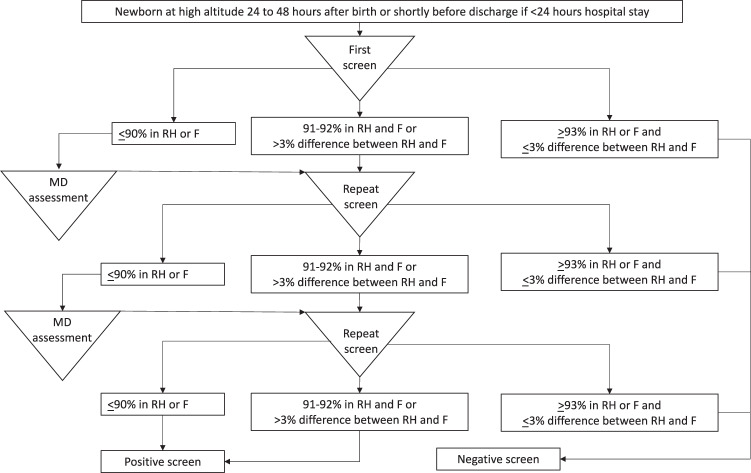
Modified high altitude oxygen saturation critical congenital heart disease screening. Modified algorithm allows for physician to determine if an echocardiogram should be obtained for oxygen saturation ≤90% before repeating the screen. However, the physician must be notified for the oxygen saturation ≤90% whereas an oxygen saturation 91–92% triggers repeat screening. RH right hand, F foot.

The SpO_2_ CCHD screening protocol at the near-sea level site during the studied period followed the AAP algorithm as outlined by Kemper et al. and consistent with the algorithm provided in the California Department of Health Care Services (DHCS) guidance provided in 2016 [2, 8]. In this algorithm, the SpO_2_ measurement was considered failing if (1) any SpO_2_ measurement was <90%, or (2) SpO_2_ 90 to <95% in both right hand and foot and/or a >3% absolute difference between the right hand and foot on three measurements. Any SpO_2_ measurement ≥95% in either the right hand or foot with ≤3% absolute difference was considered passing [2]. The SpO_2_ CCHD screening protocol at the high altitude site during the studied period used a lower SpO_2_ threshold (Fig. 1). In the high-altitude algorithm, the SpO_2_ measurement was considered failing if <93% in both right hand and foot or a >3% absolute difference between the right hand and foot on three measurements. Any SpO_2_ measurement ≥93% in either the right hand or foot with ≤3% absolute difference was considered passing. In this high-altitude algorithm, an SpO_2_ ≤ 90% prompts a physician assessment who then considers echocardiogram if other etiologies for the hypoxemia are not determined, which is similar to the AAP-Kemper algorithm [2]. In the high-altitude algorithm, the physician may recommend repeat SpO_2_ testing after the physician assessment for an SpO_2_ ≤ 90%, which is different than the AAP-Kemper algorithm.

### Statistical analysis

Our primary outcome was first-time SpO_2_ CCHD screen pass rate. We evaluated this pass rate for each sites’ screening protocol. We also evaluated the first-time pass rate for babies at high altitude using the AAP-Kemper SpO_2_ pass threshold of ≥95%. We estimated 381 newborns in each group would provide power 0.9 with alpha 0.05 to detect an increase in initial screen pass rate from 95% to 99.9%. We suspected ~60–75% of patients would have documented follow up within their birth hospital system and that not all newborns would have documented SpO_2_ numeric values. Thus, we targeted ~500 newborns in each group. Summary statistics for the newborns were presented as medians or frequencies with interquartile ranges (IQRs) or percentages, respectively. The medians of continuous data were compared using the nonparametric equality-of-medians test. The Pearson chi-square or Fisher exact test, as appropriate, was used to compare categorical data. A *p* value ≤ 0.05 was considered statistically significant. The data were analyzed with Stata Statistical Software, release 15.1 (Stata Corp, College Station, TX).

## Results

The final cohort of newborns included 463 newborns near sea level and 485 at high altitude. Demographic characteristics for the cohort are presented in Table 2. The newborns near sea level were less likely to be white (48% vs 90%, *p* < 0.001) compared to the newborns at high altitude. The newborns near sea level were more likely born via cesarean section (31% vs 24%, *p* = 0.02) compared to newborns at high altitude. The sea level newborns also had higher frequency of family history of congenital heart disease (4% vs 1%, *p* = 0.008).

**Table 2:** Demographic characteristics of newborns at sea level and high altitude.

	Sea level *N* = 463	High altitude *N* = 485	*p* value
Gender			
Female, *N* (%)	223 (48)	230 (47)	0.8
Gestational age week, median (IQR)	39 (38–40)	39 (38–40)	0.4
Race			<0.001
White, *N* (%)	224 (48)	437 (91)	
Black, *N* (%)	43 (9)	10 (2)	
Asian, *N* (%)	72 (16)	21 (4)	
Native Hawaiian or Pacific Islander, *N* (%)	10 (2)	3 (1)	
American Indian/Alaskan Native, *N* (%)	5 (1)	4 (1)	
Unknown/Not reported, *N* (%)	109 (24)	3 (1)	
Ethnicity, Hispanic, *N* (%)	130 (28)	149/480 (31)	0.04
Cesarean section birth, *N* (%)	144 (31)	118 (24)	0.02
Baby’s medical conditions			
Non cardiac defects, *N* (%)	13 (3)	12 (2)	0.8
Genetic defect, *N* (%)	1 (0.2)	2 (0.4)	>0.9
Small for gestational age, *N* (%)	23 (5)	25/481 (5)	0.9
Large for gestational age, *N* (%)	26 (6)	18/481 (4)	0.2
Neonatal respiratory condition^a^, *N* (%)	10 (2)	39/481 (8)	<0.001
Family history of CHD, *N* (%)	17 (4)	5/478 (1)	0.008

*CHD* congenital heart disease.

^a^Neonatal respiratory conditions include persistent pulmonary hypertension, transient tachypnea of newborn, respiratory distress syndrome, lung malformation, pneumothorax, meconium aspiration, and sepsis. At sea level, 8 of 10 (80%) newborns had transient tachypnea of newborn. At high altitude 36 of 39 (92%) newborns had transient tachypnea of newborn.

Medical conditions between the two groups did not differ with the exception of neonatal respiratory conditions, which were more common in the newborns at high altitude (2% near sea level vs 8% high altitude, *p* < 0.001) (Table 2). Neonatal respiratory conditions were combined to include persistent pulmonary hypertension, transient tachypnea of newborn, respiratory distress syndrome, lung malformation, pneumothorax, meconium aspiration, and sepsis. The most common neonatal respiratory illness was transient tachypnea of newborn for both groups (80% of the near sea level newborns and 92% of the high-altitude newborns with respiratory illnesses). Respiratory illnesses that presented prior to the newborn undergoing routine SpO_2_ CCHD screen were not evaluated as these newborns were excluded. The two groups were admitted to the NICU at similar rates (1% in both). Only one patient at high altitude was transferred after their routine CCHD screen, which they passed. This patient had transient tachypnea of the newborn requiring continuous positive pressure prompting the transfer.

### Oxygen saturation CCHD screen results

When applying the AAP-Kemper algorithm to all patients, high altitude patients were less likely to pass on the first SpO_2_ measurement (99.6% vs 89.3%) and more likely to require repeat screening (0.4% vs 10.1%) compared to newborns near sea level (*p* < 0.001). The adjusted high altitude SpO_2_ threshold (≥93% as opposed to ≥95% as a passing screening) resulted in 98% (*N* = 475) passing, 1% (*N* = 5) requiring repeat screening, and 1% (*N* = 5) requiring notifying the physician after their first SpO_2_ screen in the high-altitude newborns. The median preductal and postductal SpO_2_ from the first CCHD screen were lower in the high-altitude newborns (Table 3). We also evaluated how often either the first preductal or postductal SpO_2_ measurement was <95%. First SpO_2_ measurements were more likely to be <95% in the high-altitude newborns compared to newborns near sea level for both the preductal (21% vs 0.4% respectively, *p* < 0.001) and postductal (20% vs 0 respectively, *p* < 0.001) measurements.

**Table 3:** Oxygen saturation critical congenital heart disease screening results of newborns at sea level and high altitude.

	Sea level *N* = 463	High altitude *N* = 485	*p* value
AAP-Kemper^a^ algorithm applied to 1st SpO_2_ measurement, *N* (%)			<0.001
Pass	461 (99.6)	433 (89.3)	
Fail	0	3 (0.6)	
Repeat	2 (0.4)	49 (10.1)	
Number of CCHD screens completed			0.001
One, *N* (%)	461 (99.6)	468 (96.5)	
More than one, *N* (%)	2 (0.4)	17 (3.5)	
1st preductal SpO_2_, median (IQR)	99 (98–100)	96 (95–97)	<0.001
1st postductal SpO_2_, median (IQR)	99 (98–100)	96 (95–97)	<0.001
1st pre postductal SpO_2_ difference, median (IQR)	1 (0–1)	1 (0–2)	<0.001
Repeat preductal SpO_2_, median (IQR)	97 (94–99)	95 (94–96)	0.8
Repeat postductal SpO_2_, median (IQR)	98 (96–99)	96 (94–96)	0.4
Repeat pre postductal SpO_2_ difference, median (IQR)	1 (0–2)	1 (1–2)	0.7
1st preductal SpO2 less than 95%, *N* (%)	2 (0.4)	102 (21.0)^b^	<0.001
1st postductal SpO2 less than 95%, *N* (%)	0	95 (19.6)^b^	<0.001

*SpO*_*2*_ oxygen saturation, *CCHD* critical congenital heart disease, *IQR* interquartile range.

^a^AAP-Kemper algorithm result was considered failing if (1) any SpO_2_ measurement was <90%, (2) SpO_2_ 90 to <95% in both right hand and foot and/or a >3% absolute difference between the right hand and foot on three measurements. Any SpO_2_ measurement ≥95% in either the right hand or foot with ≤3% absolute difference was considered passing.

^b^Preductal or postducutal SpO_2_ < 95% differs from AAP-Kemper pass frequency because at the time the algorithm resulted in a pass as long as either the pre or postductal SpO_2_ was 95% or greater as long as the difference between the two was ≤3%.

### Confirmation of cardiac disease

To identify potentially false negative screens, follow up encounters within the medical system, at least 6 weeks after birth, were reviewed for evidence of cardiac disease. Examinations at 6 weeks of age or later were noted in the medical record for 66% of patients near sea level and 88% of high-altitude patients. No evidence of false negative screens (defined as evidence of CCHD in a newborn that passed SpO_2_ screening) was found in either group. The two groups had echocardiograms completed at similar frequencies, 2.8% (*N* = 13) of patients near sea level and 1.9% (*N* = 9) of high-altitude patients (*p* = 0.3). Of the patients that had echocardiograms, 56% of high-altitude patients (5 of 9) had abnormal echocardiograms while 23% of patients at sea level (3 of 13). The differences in abnormal echocardiograms were not significant (*p* = 0.12). Patent ductus arteriosus and/or patent foramen ovale were not considered abnormal. The echocardiogram abnormalities were ventricular septal defects (*N* = 7), and mild pulmonary hypertension (*N* = 1). We performed a secondary analysis restricting our patient population to those with a documented follow up examination at 6 week of age or older. Even after this restriction, the high-altitude patients were still more likely to require repeat SpO_2_ measurement after the first measurement compared to patients near sea level (0.3% vs 11%, *p* < 0.001) when using the AAP-Kemper algorithm. None of the infants included in the study underwent cardiac catheterization or cardiac surgery at the Regional Perinatal Center in the first month after birth.

## Discussion

Lowering the SpO_2_ CCHD passing threshold to ≥93% increases the frequency of first screen pass among newborns at high altitude (>6000 ft). When using a SpO_2_ threshold ≥95%, less than 90% of newborns at high altitude passed their first screen. Decreasing the pass threshold to ≥93% resulted in 98% of newborns at high altitude passing on the first CCHD screen. This is not surprising considering the median preductal and postductal SpO_2_ results were 96% in the high-altitude patients in our cohort. We obtained follow-up data for 88% of the high-altitude patients after at least 6 weeks of age and did not find evidence of a missed CCHD in a patient that passed the SpO_2_ screen, or in other words, we did not find evidence of a false negative screen.

The SpO_2_ thresholds implemented in initial AAP-recommended CCHD screening algorithm were based on studies of thousands of newborns, including newborns both with and without CCHD [2, 9, 10]. Since then, universal SpO_2_ screening has improved early detection of CCHD and decreased mortality [11]. SpO_2_-based CCHD screening has also been noted to have a small false positive rate at 0.14% overall and 0.06% if performed 24 h after birth or later [3]. However, it is notable that the recommended SpO_2_ thresholds were based on studies on newborns predominantly at lower altitude [9, 10]. Furthermore, ways to further improve the algorithm, including at higher altitude, have been noted [12]. Hospitals at high altitude have noted increased false positive rates using the standard AAP SpO_2_ thresholds leading to a significant increase in the number of unnecessary echocardiograms required [4, 5]. Considering the most recent updated CCHD algorithm now only requires one repeat measurement as opposed to two before classifying as a failed screen and potentially triggering an echocardiogram, the pass threshold at high altitude is even more crucial to clarify [12]. In theory, in this 2020 recommended algorithm, using the standard pass threshold at higher altitude could result in a larger overall screen fail rate as the newborns would have fewer opportunities to pass the screen.

In Table 4, we provided a summary of prior studies that made adjustments to the screening algorithm at higher altitude and will discuss some of them further here [4, 5, 13, 14]. Some centers at high altitude, such as Aurora CO, have altered their algorithms by using a cut-off of <85% for a positive screen (instead of <90% in the AAP algorithm) and ≥90% with <3% preductal postductal difference on repeated attempts as a screen negative (instead of ≥95% in the AAP algorithm) [4]. However, in this algorithm first screens still require ≥95% in the preductal or postductal SpO_2_ on the initial screens to pass without needing to repeat the measurement [4]. In another modified high altitude algorithm, the initial and overall SpO_2_ passing thresholds also remain ≥95% [14]. For example, some hospitals in Colorado have made modifications such as delaying the screen to 30 h after birth to allow further transitioning, lowering the SpO_2_ failure threshold to <85%, and trialing oxygen hood to increase PIO_2_ for 20 min for those requiring repeat screens [4, 6, 14]. These algorithms however still require SpO_2_ ≥ 95% to pass the screen. Therefore, these adjustments may lower the overall false positive rate as noted by refs. [4, 14] (Table 4). However, they may still require additional nursing time to repeat screening measurements. The non-passing rate of the first screen in these studies was 5.8% and 3.6%, much higher than the non-passing rate of newborns at sea level or newborns at high altitude with the threshold of ≤93% in our cohort (2%). The modified high-altitude screening thresholds described in our study allowed for a significant reduction in the number of patients requiring repeat screening measurements from 10.1% to 2%.

**Table 4:** Summary of critical congenital heart disease screening approaches at high altitude.

	Altitude and study size	Adjust timing of screen	Failure threshold	Pass or repeat thresholds^a^	Trial of oxygen	Non-passing 1st screen rate	Overall failed (or positive) screen	False Positive
Wright et al. [4]	5557 ft *N* = 1003	No	Lowered to <85%	Range widened to 85–94% requiring a repeat test, the pass threshold remained ≥95%	No	5.8%	1.1%	unknown
Lueth et al. [14]	6200 ft *N* = 2001	No	Lowered to ≤85% for 1st screen; remained <90% for repeat screens	Range widened to 86-94% requiring repeat for the 1st screen, additional screens remained at 90–94% to prompt repeating, the pass threshold remained ≥95%	Yes if 1st screen 86–94% or difference >3%, trial of 26% F_I_O_2_ hood for 20 min before rescreening on room air	3.6%	0.3%	﻿Echocardiograms were performed on four of six failing newborns (two newborns who went on to pass additional screens were considered to have passed and did not have imaging). Of the four failing newborns, all had normal anatomy. No CCHD was identified.
Paranka et al. [5]	24 sites <2000 ft *N* = 4101 5 sites 4700–6000 ft *N* = 1387 5 sites >6000 ft *N* = 656	No	Remained <90%	One high altitude site lowered threshold to ≥93% instead of ≥95% to pass			0.2% <2000 ft 1.2% 4700–6000 ft 6% >6000 ft	44 of the 65 positive screen patients underwent echocardiograms, of which 91% were normal. The false positives occurred nearly all above 4700 ft, 36 of 40 (90%) of false positives.
Rao et al. [13]	*N* = 3548 5400 ft	No	Remained <90%	Remained 90–94% to prompt repeat and ≥95% to pass	No	Data not provided. However, at least 7.7% received at least one repeat screen (273/3548 passed on 3rd attempt)	Data not provided	2.6% false positives identified. Rate decreased from 3.5% to 1.5% with time but remained over that of the sea-level (0.035%)

^a^Right hand-to-foot difference ≤3% remained a passing threshold and difference >3% prompted a repeat screen in all studies.

Our first non-pass rates when using the AAP threshold ≥95% are higher than some prior studies. For example, 10.1% of our high-altitude patients did not pass the first screen with this standard threshold, which is higher than the non-pass rates reported by Wright et al. and Lueth et al. at similar altitude ~5000–6000 ft [4, 14]. Our higher non-pass on the first screen is likely due to our retrospective design versus their prospective and possibly more controlled approach, or due to site-to-site variation. A multicenter study of various altitudes conducted by Paranka et al., also showed an increase in the positive screen rate with increasing altitude. However, when using the AAP-Kemper passing threshold of ≥95%, only 6% of newborns >6000 ft had a positive screen [5]. Our higher false positive rate compared to Paranka et al. findings are likely due to us only evaluating the first SpO_2_ measurement as opposed to the overall screening algorithm. If our patients had proceeded onto repeat measurements, then the overall positive screen rate presumably would have decreased.

Our findings are consistent with physiologic considerations at high altitude in newborn infants (Table 1). Increasing altitude reduces barometric pressure and PIO_2_. Calculations based on barometric pressure data demonstrate a 27 mmHg reduction in Alveolar PAO_2_ at 6000 ft altitude compared to sea level. The Alveolar to arterial gradient (A-a gradient) is higher in healthy newborn infants compared to adults and is reported to be 28.3 ± 10.1 mmHg [15]. However, in adult models, the A-a gradient decreases at high altitude and a similar mechanism can be expected in neonates resulting slightly higher PaO_2_ values than expected based on drop in alveolar PAO_2_ (Table 1) [16]. Presence of fetal hemoglobin and high respiratory rates, leading to alkalosis, shift the oxygen-hemoglobin curve to the left resulting in higher SpO_2_ for a given PaO_2_ [17, 18]. For these physiological reasons, neonates at high altitude tend to have relatively higher SpO_2_ despite low PaO_2_.

Interesting that despite difference in access to a NICU, the two sites had similar admission rate to the NICU (1%). The site near sea level has a level IV NICU within the same building whereas the high-altitude sites does not have a local NICU and the closest NICU is either a flight or drive on a mountainous road away. Thus, we expected that the well newborn nursery at the high-altitude site may manage higher acuity patients compared to the near sea level site to avoid a transfer. This may still be the case though, as we excluded patients that were transferred to the NICU prior to their routine SpO_2_ screen or if the SpO_2_ was measured prior to 24 h of age due to symptoms rather than early discharge, in order to isolate only the SpO_2_ measurements done purely for screening in asymptomatic infants. Despite difference in access to echocardiograms between the two sites, echocardiograms were also obtained at similar rates at the two sites despite differences in screen pass rates. Our approach was limited to review the indication for echocardiograms, but we suspect the majority were done due to other clinical indications such as findings of murmur on physical examination.

There are several limitations to our study. As a retrospective study with a total sample size of 948 patients, we are limited by the documentation in the chart and limited ability to estimate false negative screen rates. Due to the low incidence of CCHD we were unable to identify any CCHD cases in our population. This could also be due to the improved prenatal detection of CCHD cases that led to prompt NICU admissions at birth and exclusion from our study cohort. This is consistent with similar CCHD screening studies that were unable to identify cases of CCHD in their cohort [19–21]. Hence, we could not confirm the effect of the altered CCHD screening protocol on false negative rates. Additionally, we were unable to review birth defect registries for possible false negative cases as the California Birth Defects Monitoring Program Registry monitors ten counties (30% of births in California). The studied population was not in one of those monitored counties [22]. We were however able to confirm follow up and absence of concern for congenital heart disease in 88% of the patients at high altitude. Additionally, there are only two major cardiac centers within California that are near the high-altitude center, of which our near sea level site is one of them and due to partnership between the two sites it is most likely a patient would have been transferred to our center. We were also limited by the SpO_2_ values actually performed. Thus, when applying the AAP-Kemper algorithm to the patients at high altitude, we were not able to assess the overall false positive rate of the algorithm as the patients did not always have a repeated measurement that would have been triggered by that algorithm. None the less, our findings that an altered pass threshold reducing the need for repeat measurements is important. Additionally, in the context of recent recommendations that reduce the number of repeat screens before considering a screen as a failed screen, this should be evaluated further specifically at higher altitude.

In conclusion, lowering SpO_2_ CCHD pass threshold by just 2% (from ≥95% to ≥93%) at 6000 ft, significantly increased pass rate on first screen. It also does not appear to increase false negative screens; however, further studies with larger samples will be needed to identify missed CCHD cases and should evaluate the diagnosis of other diseases with hypoxemia as well (i.e., persistent pulmonary hypertension of the newborn). Adjusting the CCHD pass threshold may reduce nursing time associated with unnecessary repeat measurements and may reduce overall healthcare spending due to avoidable echocardiograms. It can also prevent unnecessary transfers to tertiary hospitals. Therefore, an altered screening protocol at high altitudes may reduce parental anxiety of a failed screen due to the additional hospital length of stay and the prohibitive costs of additional diagnostics. Our findings suggest altitudes 5001–7500 ft could consider reducing the threshold to ≥93% similar to our study. However, larger samples are needed to confirm these findings. Using larger samples, it might be possible to come up with a simple algorithm (such as reducing the threshold from 95% by 1% for every 1000 m above sea level). The risk of false negatives with modifications to the thresholds needs to be considered and studied as well. If the passing thresholds for CCHD screening do not change at higher altitude due to risk of false negatives, then other mitigation efforts such as improved local access to echocardiogram should be considered. We recommend that individual high-altitude centers evaluate their CCHD screening algorithm and publish their data to enable AAP and CDC to come up with new algorithms for high-altitude screening.

## Data Availability

The datasets generated and analyzed during this study are not publicly available through a repository but are available from the corresponding author on reasonable request and may require institutional data agreements.
